# Optimization of Translation Profiles Enhances Protein Expression and Solubility

**DOI:** 10.1371/journal.pone.0127039

**Published:** 2015-05-12

**Authors:** Anne-Katrin Hess, Paul Saffert, Klaus Liebeton, Zoya Ignatova

**Affiliations:** 1 Institute of Biochemistry and Biology, University of Potsdam, Potsdam, Germany; 2 BRAIN, Zwingenberg, Germany; 3 Biochemistry, Department of Chemistry and Biochemistry, University of Hamburg, Hamburg, Germany; Tel Aviv University, ISRAEL

## Abstract

mRNA is translated with a non-uniform speed that actively coordinates co-translational folding of protein domains. Using structure-based homology we identified the structural domains in epoxide hydrolases (EHs) and introduced slow-translating codons to delineate the translation of single domains. These changes in translation speed dramatically improved the solubility of two EHs of metagenomic origin in *Escherichia coli*. Conversely, the importance of transient attenuation for the folding, and consequently solubility, of EH was evidenced with a member of the EH family from *Agrobacterium radiobacter*, which partitions in the soluble fraction when expressed in *E*. *coli*. Synonymous substitutions of codons shaping the slow-transiting regions to fast-translating codons render this protein insoluble. Furthermore, we show that low protein yield can be enhanced by decreasing the free folding energy of the initial 5’-coding region, which can disrupt mRNA secondary structure and enhance ribosomal loading. This study provides direct experimental evidence that mRNA is not a mere messenger for translation of codons into amino acids but bears an additional layer of information for folding, solubility and expression level of the encoded protein. Furthermore, it provides a general frame on how to modulate and fine-tune gene expression of a target protein.

## Introduction

Gene expression is extensively regulated at different levels, including transcription, mRNA degradation, translation and protein degradation [1]. Translation, as the most downstream process in gene expression, provides the necessary plasticity to fine-tune protein levels [2]. Several factors regulate the expression of a gene at the level of translation, including global mRNA secondary structure [3], local mRNA structure around the initiation site [4–6], mRNA half-life [7, 8], tRNA concentration of the expression host [9–11], ribosome loading [12, 13] and recycling [14], and interactions between the nascent chain and ribosome [15]. Initiation of translation is typically viewed as the main regulatory element of translation particularly for mRNAs with stable 5’ secondary structure [16, 17]. However for subset of messages with a weaker secondary structure or by mRNA excess over the free initiating ribosomes (e.g., in some stress conditions), the bottleneck in protein production shifts to elongation [18–20]. Non-uniform elongation speed cooperates with processes downstream of translation and maximizes the yields of soluble, functionally-active protein [12, 21–24]. Importantly, changes in the speed with which ribosomes read an mRNA render the protein insoluble [9, 23, 25–27]. Clearly, the non-uniform elongation speed is beneficial for the expression of endogenous proteins, but the effect of non-uniform translation on the expression of heterologous proteins is still unclear.

Usually, highly expressed genes are enriched in codons that are read by abundant tRNAs [28]. Thus, a commonly used strategy to facilitate an expression of a recombinant protein is to synonymously substitute all codons to rapidly translated ones, i.e. to ones read by high-abundance tRNAs. Although successful for single-domain proteins, such a strategy would eliminate all slow-translating regions that synchronize translation with co-translational folding of a multidomain protein [27]; elimination of translational pauses renders the protein misfolded [9]. Furthermore, using strains with altered ribosomes that globally translate slowly proves to be efficient in expression of some proteins [29], but the extremely slow growth of the strain is often problematic. Similarly, growth of the host at lower temperatures, which also globally slows down translation, improves the functional expression of proteins [30] by increasing the time window for co-translational folding of each domain [27]. Alternatively, low-induction regimes decrease the production of protein amount per unit of time which allows operating below the aggregation threshold of the protein and improve the expression of soluble protein [31]. Although effective in enhancing the amount of functional folded protein, the global yields, however, remain low.

Adaptation of the translation profile of a heterologous gene to the tRNAome of the expression host has more potential to enhance its expression [32] but requires knowledge of the tRNA concentration of both the native and expression strains. The tRNA concentration is only known for three species so far, *E*. *coli* [33], *Bacillus subtilis* [34] and *Lactobacillus lactis* [35]. Sequencing-based approaches to determine the tRNAome of unicellular and multicellular organisms are suggested [36], however the bulky nature of some tRNA modifications puts into question the precision of quantification methods based on hybridization or RT-PCR. Importantly, the codon reading program can be dramatically altered upon amino acid starvation [37]. In multicellular organisms, despite the identical genomic tRNA repertoire, the concentration of isoacceptor tRNAs for a set of synonymous codons varies among tissues and stages of differentiation [38]. Furthermore, the evolutionarily selected optimal codons in some bacteria do not match the genomic tRNA copy numbers [39] which might be explained by species-specific, developmental stage-specific modifications [39–41] or even nutrient-driven alterations in tRNA modifications [41]. How can translation pattern be adapted without knowledge of the tRNA concentration and modification pattern within the parental organism?

Here we developed a structure-based approach to deduce an optimal translation profile for a gene whose native translation pattern in the parental strain is unknown. In the core of this approach is the observation that structurally and functionally-related proteins from different organisms share similar translation profiles [42]. Introducing slow-translating regions that delineate the structural domains in epoxide hydrolases (EHs), used as model proteins, largely increased their solubility, most likely by facilitating their co-translational folding. Furthermore, we introduced synonymous substitutions to decrease the folding energy of the initial 5’-coding region of one EH which enhanced the expression level by facilitating translation initiation. In general, our results imply that synonymous alterations of mRNA to adapt its translation profile to the tRNA household of the expression host and to improve initiation by alterations of the folding energy in the initial 5’ coding region are successful strategies to fine-tune the expression of heterologous proteins.

## Experimental Procedures

### Databases and bioinformatic tools

Protein-coding sequences were retrieved from the NCBI GenBank Database [43] and the secondary structures were predicted with PredictProtein[44] (https://www.predictprotein.org/). Subsequently, homologous protein 3D structures were revealed by comparison of the coordinates retrieved from PDB files using the Dali Server [45] (http://www.bioinfo.biocenter.helsinki.fi/dali_server/start), whereas secondary structures of sequences with unknown crystal structures were aligned with PROMALS3D server [46]. (http://prodata.swmed.edu/promals3d/promals3d.php). Translation profiles were generated using RiboTempo [42] (http://www.chemie.uni-hamburg.de/bc/ignatova/tools-and-algorithms.html) which uses the experimentally determined tRNA concentrations of *E*. *coli* [33]. The mRNA folding energy profiles were generated using the RNAfold program from the Vienna RNA Package [47] (http://rna.tbi.univie.ac.at/cgi-bin/RNAfold.cgi) with default parameters. The Gibbs free energy was calculated with a 39 nt-sliding window [4].

### Protein constructs, expression and cell fractionation

A plasmid containing the *echA* gene from *A*. *radiobacter* was kindly provided by D. Janssen [48] and the genes of all other EHs were derived from metagenomic libraries from B.R.A.I.N AG collection (M5bG7, M9dH11) [49]. EHs encoding genes were cloned in pBAD/Myc-His A (amp^R^) under the control of L-arabinose inducible P_BAD_ promoter. We synonymously exchanged codons by site-directed mutagenesis to remove translational attenuation sites of the *echA* gene (Wt, LHH, LS, LL, All), and introduce translational attenuation sites to M5bG7 (M5Wt, M5-L1, M5-L2, M5-LL) and M9dH11_opt (M9Wt_opt, M9-L1_opt, M9-L2_opt, M9-LL_opt). The third base of the initial first codons of M9dH11 (ATGAAACCCCGCACGGTGCCG) was modified to A in M9dH11_opt (ATGAAACCACGAACAGTACCA) without changing the protein sequence (MKPRTVP) to decrease the folding energy of this initial region.

All variants were expressed in *E*. *coli* BL21(D3) cells grown at 37°C in LB medium supplemented with 100 μg/mL ampicillin. Protein expression was induced with 0.2% arabinose at OD_600_ ≈ 0.5 (±0.1) and cells were harvested 1–2 h (1h for EH-Ar and M5bG7 mutants and 2 h for the M9_opt variants) post-induction dependent on the EH construct. For total protein expression analysis, aliquots of cells were harvested, lysed in SDS-PAGE loading buffer, heated to 100°C for 5 min and treated with Benzonase (Sigma-Aldrich) for 30 min on ice and subsequently analyzed by SDS-PAGE, immunoblotting or subjected to RNA isolation. Proteins were detected with a monoclonal mouse-α-His_6_ antibody (Calbiochem). GAPDH was used as a loading control and immunostained with a polyclonal goat-α-GAPDH (Genescript).

For cell fractionation, cell aliquots of 6 OD_600_ units were rapidly cooled on ice and then harvested by centrifugation at 4000xg for 15 min at 4°C. The cell pellet was resuspended into 120 μL lysis buffer A (10 mM potassium phosphate pH 6.5, 1 mM EDTA, 20% sucrose, 1 mg/mL lysozyme) and incubated for 30 min on ice. 1080 μL of buffer B (10 mM potassium phosphate pH 6.5, 1 mM EDTA) was added prior to sonication (Digital Sonifier S-250D microtip, Branson) for 1 minute at 65% (4 sec pulse, 11 sec pause). Intact cells and cell debris were removed by centrifugation at 2000xg for 20 min at 4°C. The supernatant was transferred equally into two fresh tubes (600 μL each). One sample was not further treated and considered as total protein sample. The other sample was used to separate soluble and insoluble proteins via centrifugation at 15000xg for 20 min at 4°C. The supernatant was kept as it contains the soluble proteins. The pelleted insoluble proteins were resuspended in 600 μL of lysis buffer B by brief sonication (few seconds, 20%) and centrifuged at 15000xg for 20 min at 4°C. The pellet was again washed with 600 μL lysis buffer B by brief sonication and centrifuged at 15000xg for 30 min at 4°C. Finally the pellet was resuspended in 600 μL of lysis buffer B. Total, soluble and insoluble protein fractions were analyzed by SDS-PAGE and Western blot.

### Total RNA isolation and quantitative RT-PCR

Total RNA was isolated from 1 mL cells in 1 mL TRI reagent (Sigma-Aldrich) according to the manufacturer’s protocol. The centrifugation steps after isopropanol precipitation and washing with ethanol were extended to 30 min at 21,000xg at 4°C. The RNA pellet was dissolved in RNAse-free water and the RNA quality was verified by the absorbance ratio A260nm/A280nm (≥ 1.8) and agarose gel electrophoresis.

RNA samples were subjected to DNase I (Thermo Scientific) treatment according to the manufacturer’s protocol. cDNA was produced by reversed transcription with RevertAid H Minus (Thermo Scientific) using random hexamer primers. mRNA levels were quantified by qRT-PCR using QuantiFast SYBR Green PCR Kit (Qiagen). For each primer pair a no-template control and a no reverse transcriptase control were performed. The level of mRNA was normalized to the level of GAPDH mRNA as an internal standard.

### Calculation of the folding energy

The Gibbs free energy was calculated for each sequence from nucleotide position -50 to +50 with a sliding window of 39 nt [4], and this value was assigned to the nucleotide position of the window centre. We used the Vienna RNA Package, version 1.8.5, available at http://www.tbi.univie.ac.at/%7Eivo/RNA/, to predict free energy of RNA sequences.

## Results

### Translational attenuation sites in EH from *A*. *radiobacter* influence its solubility in *E*. *coli*

**Fig 1:**
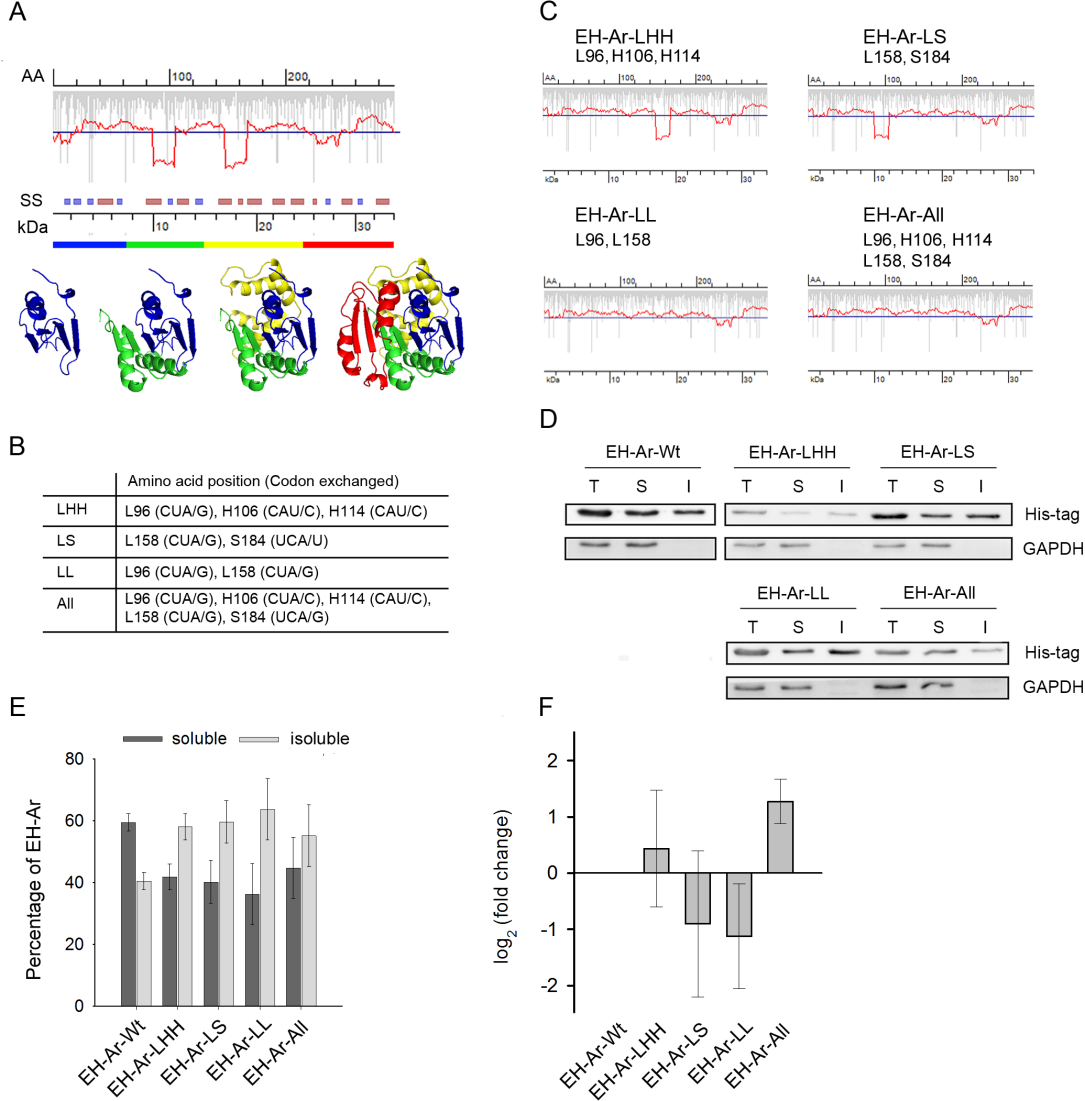
Translation attenuation sites delineate domain boundaries and impact protein solubility. (A) Translation profile of EH-Ar predicted with RiboTempo. Vertical gray bars represent the rate of translation of each single codon which is averaged (red line) along the whole ORF with a window of 19 codons [42]. Translation minima below the genome-wide threshold (blue horizontal line) denote the putative slow-translating attenuation sites. AA denotes amino acid number, kDa marks the corresponding molecular weight and SS denotes the predicted secondary structure (β-sheets—blue bars, α-helices—dark red bars, uncolored empty space—linking structural elements). The rainbow-colored bar visualizes the putative structural domains, colored in the same way in the 3D- structure (PDB 1EHY). (B) Summary of the exchanged codons in EH-Ar. The position of the exchanged amino acids for each variant is indicated. (C) Translation profiles of EH-Ar variants with exchanged slow translating patches (B) predicted with RiboTempo. (D, E). Removal of the translational attenuation sites reduces the solubility of EH-Ar. (D) Representative immunoblot of EH-Ar variants (summarized in C). The total (T) protein content was fractionated into soluble (S) and insoluble (I) fractions and 0.05 OD_600_ of cells were applied per lane. GAPDH served as a loading control; note that it is a completely soluble protein and its absence in the insoluble fraction confirms the good quality of the fractionation procedure. (E) Quantification of the immunoblots of three biological replicates ± SEM. Each total fraction was normalized to GAPDH intensity to allow for comparison between the samples; the soluble and insoluble fractions were determined as a percentage of this normalized value. *, p<0.05, Tukey’s test. (F) Quantification of mRNA levels by qRT-PCR of the EH-Ar variants. Values were normalized to GAPDH mRNA expression, represented as a fold-change to the wild-type mRNA and are means ± SEM (n = 3).

EHs catalyze the conversion of epoxides to their corresponding diols and play a major role in the detoxification of chemically reactive molecules [50]. Many attempts to recombinantly express EHs from various sources in *E*. *coli* have typically resulted in the formation of inclusion bodies that are composed of insoluble and misfolded protein [50]. *E*. *coli* does not encode any EH to assess a translation profile of endogenous enzyme whose expression would be evolutionarily optimized for the *E*. *coli* tRNAome. EH from *A*. *radiobacter* (EH-Ar) exhibits the highest solubility in *E*. *coli*; it partitions to approximately 50% in the soluble fraction [48]. Thus, we reasoned that the EH-Ar translation profile in *E*. *coli* should at least in part resemble the translation profile of EH-Ar in its parental strain. Using RiboTempo we calculated the EH-Ar translation profile in *E*. *coli* which revealed three minima positioned downstream of structural domains (Fig 1A). Importantly, the minima are located approximately thirty amino acids downstream of the domain boundaries, which is a common length of nascent peptide protected by the ribosome within the exit tunnel [51]. Therefore, slow translation is synchronized with emergence of the structural domain from the ribosome. To determine whether these putative slow-translating regions influence the co-translational folding and consequently the solubility of EH-Ar, we substituted all slow-translated codons in these patches [42], here Leu, His and Ser codons (Fig 1B), with synonymous codons read by the most abundant tRNA species in either of the first two deep minima (EH-Ar-LHH at approximately 10 kDa and EH-Ar-LS at approximately 18 kDa, Fig 1B and 1C), or in both (EH-Ar-all, Fig 1B and 1C). The EH-Ar-All variant represents a sequence translated at nearly uniform, fast elongation speed (except the third shallow minimum, Fig 1B and 1C). All variants were expressed in *E*. *coli* BL21(DE3), and the partition between soluble and insoluble fractions was analyzed (Fig 1D and 1E). Notably, the local acceleration of translation, by disruption of any of the translation minima, increased the amount of the protein found in the insoluble fraction and the solubility of all constructs decreased from approximately 60% (wild-type) to 40% (Fig 1D and 1E).

The Leu CUA codon has one of the rarest tRNAs in *E*. *coli* [33] and it is present in both deep minima of EH-Ar. Hence, we also created a variant in which those two single Leu CUA codons were replaced with CUG (EH-Ar-LL, Fig 1B and 1C). Interestingly, even the substitution of both Leu CUA codons in the EH-Ar-LL construct was sufficient to considerably reduce the EH-Ar solubility (Fig 1D and 1E). The mRNA expression levels of all variants were similar to the wild-type EH-Ar as revealed by quantitative RT-PCR and cannot account for the observed variations in the solubility of the EH-Ar variants (Fig 1F). We used a low copy plasmid whose expression is much closer to the single-copy chromosomal expression of natural *E*. *coli* genes. Notably, high copy plasmids producing high transcript level of the target mRNA may cause a disbalance in the tRNA equilibrium of the host cell through the increased demand for specific tRNAs. Consequently, it may alter the natural codon usage bias of the host [52] and the rate of translation of each single codon; thus, high copy plasmids may not represent the natural expression levels in *E*. *coli*. Together our results show that the solubility of EH-Ar in *E*. *coli* is strongly influenced by slow-translating regions that delineate structural domains and likely facilitate co-translation in a manner similar to endogenous proteins [27].

### Introduction of translation attenuation sites renders insoluble EHs soluble

**Fig 2:**
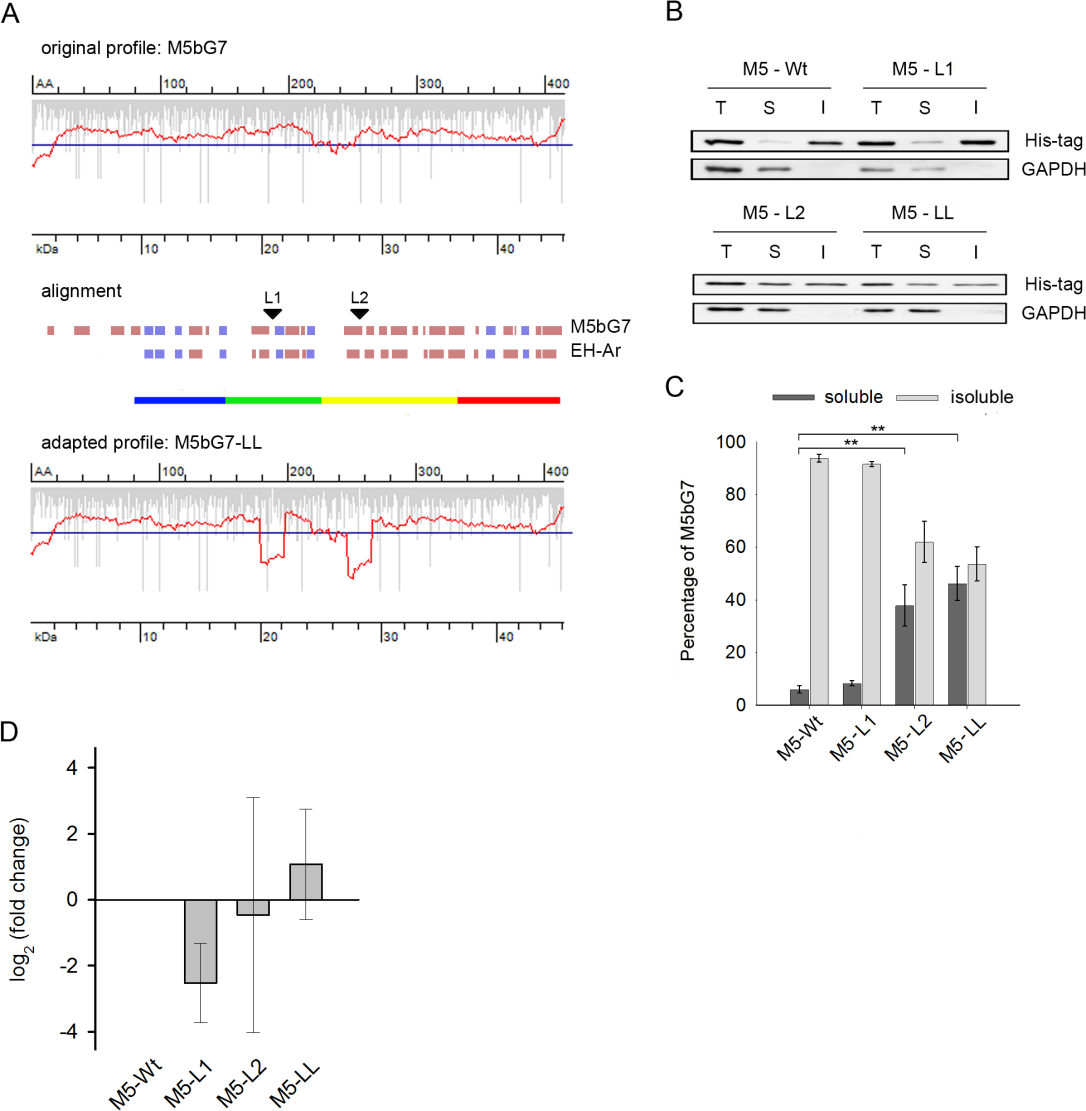
Introducing translational attenuation sites into the EH M5bG7 sequence enhances its solubility. (A) Translation profile of wild-type M5bG7 (top diagram, original profile) and upon introduction of translational attenuation sites (bottom diagram, adapted profile) in *E*. *coli*. Secondary structure alignment (β-sheets—blue bars, α-helices—dark red bars, uncolored empty space—linking structural elements) of M5bG7 to EH-Ar to identify regions for introduction of slow-translating stretches (indicated with arrows). The domains in M5bG7 were delineated based on the domain architecture of EH-Ar represented in the color code as in Fig 1A. Note the longer N-terminal domain of M5bG7 than that of EH-Ar. (B) Representative immunoblot of M5bG7 (abbreviated M5) variants. L1 (Leu 189, CUC/A, numbering is according to the M5bG7 sequence) and L2 (Leu 257, CUG/A) denote the synonymous exchange of a fast-translating Leu codons to Leu CUA in the first and second attenuation, respectively, and LL (Leu189, CUC/A, Leu257, CUG/A) in both simultaneously. T, total protein, S, soluble and I, insoluble fraction. GAPDH served as loading control. (C) Quantification of immunoblots of three biological replicates ± SEM. **, p<0.01, Tukey’s test. For details refer to the legend of Fig 1. (D) Quantification of mRNA levels by qRT-PCR of the M5bG7 variants. Values were normalized to GAPDH mRNA expression, represented as a fold-change to the wild-type mRNA and are means ± SEM (n = 3).

**Fig 3:**
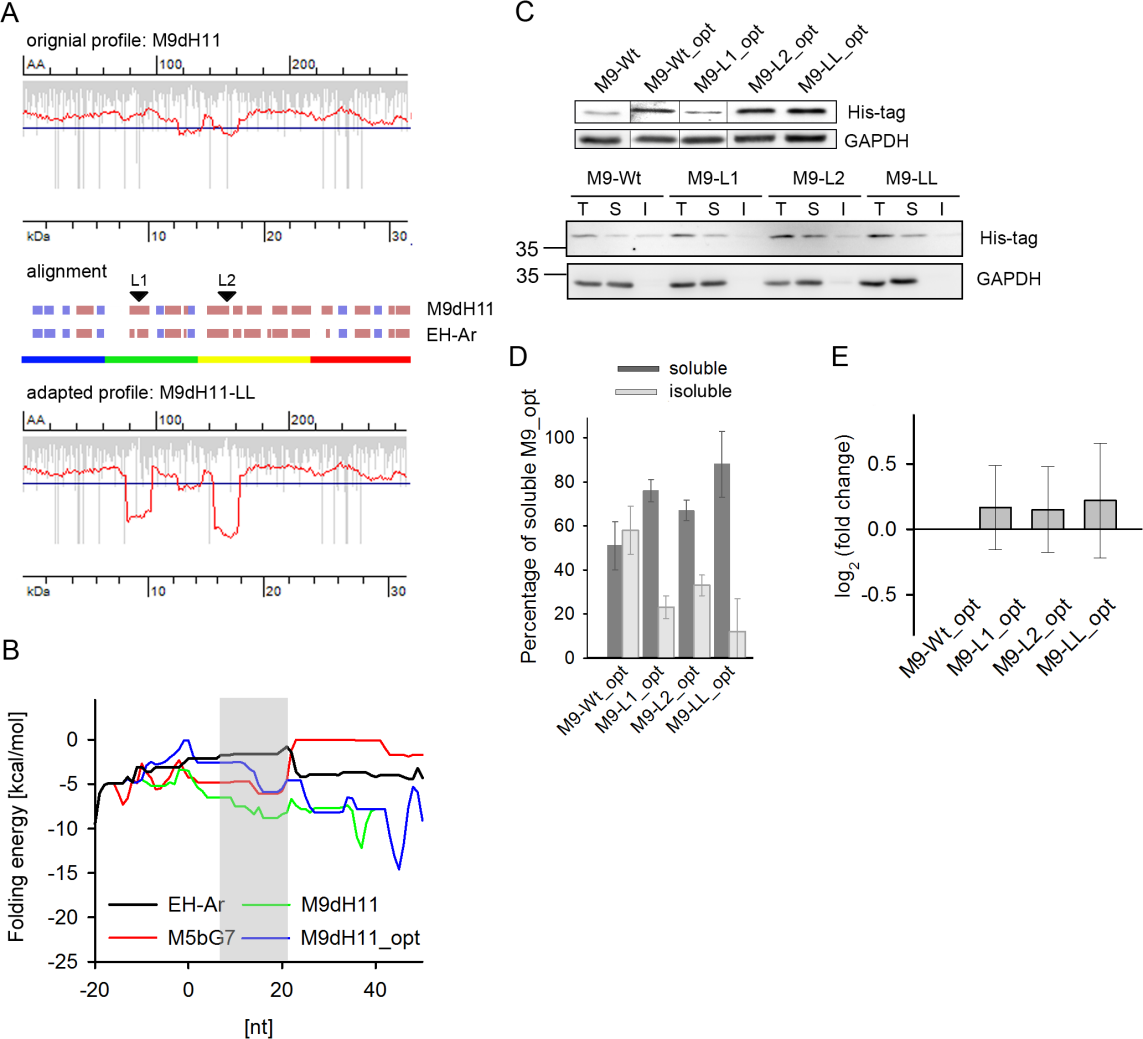
Introducing translational attenuation sites into the EH M9dH11 sequence enhances its solubility. (A) Translation profile of wild-type M9dH11 (top diagram, original profile) and upon introduction of translational attenuation sites (bottom diagram) in *E*. *coli*. Secondary structure alignment (β-sheets—blue bars, α-helices—dark red bars, uncolored empty space—linking structural elements) of M9dH11 to EH-Ar to identify regions for introduction of slow-translating stretches (indicated with arrows). The domains in M9dH11 were delineated based on the EH-Ar domain architecture presented in the color code used in Fig 1A. (B) Local folding energies of the mRNA sequences of EH variants around the start codon. The zero nucleotide position corresponds to first nucleotide of the start ATG codon. The shadowed area marks the codons (3^rd^ to 7^th^), at which changes were undertaken in M9dH11_opt. The -50 nucleotides for all four sequences are identical and determined from the upstream region of the expression vector. For comparison, two EH variants, EH-Ar and M5bG7, with lower folding energies than that of M9dH11 are included. The energy of M9dH11_opt falls between that of EH-Ar and M5bG7. (C) Representative immunoblot (n = 3) of total expression (upper panel) of M9dH11 (abbreviated M9) variants and fractionation to soluble and insoluble fractions (bottom panel). M9_opt denotes the variants with optimized secondary structure at the 5’-end. L1 (Leu 88, CUG/A, numbering is according to the M9dH11 sequence), L2 (Leu154, CUG/A) and LL (Leu88, CUG/A, Leu154, CUG/A) denote synonymous exchange of a fast-translating Leu codon to Leu CUA in the first, second or both attenuation sites (LL), respectively. T, total protein, S, soluble and I, insoluble fraction. GAPDH served as loading control. For details refer to the legend to Fig 1. (D) Quantification of immunoblots of two biological replicates ± SD. (E) Quantification of mRNA levels by qRT-PCR of the M9dH11_opt variants. Values were normalized to GAPDH mRNA expression, represented as a fold-change to the wild-type mRNA and are means ± SEM (n = 3).

Based on the EH-Ar example, we hypothesized that synchronization of the translation profiles of other insoluble EHs with that of EH-Ar will facilitate their soluble expression in *E*. *coli*. We took two different EHs, M5bG7 and M9dH11 of metagenomic origin [49], which partition mostly in the insoluble fraction when expressed in *E*. *coli*. Their translation profiles in *E*. *coli* were predominantly smooth (Figs 2A and 3A, original profiles). EHs display highly conserved three-dimensional structures with a common α/β hydrolase fold [53]. To determine the structural domains in M5bG7 and M9dH11, we aligned their predicted secondary structures to the EH-Ar secondary structure (Figs 2A and 3A, alignments). Notably, the secondary structure of the three EHs revealed a conserved pattern which aligned to the domain structure of EH-Ar was used to delineate the single domains of M5bG7 and M9dH11. In EH-Ar the slow-translated regions are located approximately 30 amino acids downstream of the domain boundaries (Fig 1A), hence for introducing the slow-translating regions in M5bG7 and M9dH11 we selected regions at a similar distance from the C-termini of their putative domains. Thus, the pausing sites were positioned downstream of the domains boundaries including a stretch of the peptide chain that will be covered in the ribosomal tunnel. M5bG7 possesses an additional N-terminal domain (Fig 3A) which is absent in the other EHs; this difference was considered by the positioning of the translational attenuation sites. Interestingly, in all regions we selected for introducing the slow-translating codons Leu residues are present, although those Leu residues in M5bG7 and M9dH11 they are encoded by fast-translating Leu codons as compared to the slow-translating CUA codon in EH-Ar. Since for EH-Ar even a single CUA codon was sufficient to confer a transient pause (Fig 1A, EH-Ar-LL mutant), to create variants with slow-translating patches in each selected region downstream of the putative domains in M5bG7 and M9dH11 we substituted only one Leu-encoding triplet with the CUA codon (Figs 2A and 3A, adopted profiles). In M5bG7 the introduction of the first attenuation site (M5-L1 mutant) did not alter its solubility, while the second attenuation site (M5-L2 mutant) enhanced the solubility of M5bG7 to approximately 40% (Fig 2B and 2C). Since no activity test is available for these enzymes to probe their physiologically active, folded structure, we used solubility in the cytosol of the expression host as a proxy of folded structure. None of the synonymous mutations altered the mRNA level compared to the wild-type M5bG7 (Fig 2D), thus the observed effect in the solubility was solely a result of changes in the translation speed.

In contrast to the high expression levels of wild-type M5bG7 in the insoluble fraction, wild-type M9dH11 was expressed at very low levels at the detection limit of immunoblot. Thus, we first sought to enhance the total yield of M9dH11 to be somewhat comparable to M5bG7. For the M9dH11 expression, we used the same expression vector as for EH-Ar and M5bG7 so that their expression is controlled by identical promoter and translation initiation-controlling elements (e.g., Shine-Dalgarno-sequence and 5’ UTR region). We hypothesized that the low expression of M9dH11 could be due to initiation interference by mRNA structure in the vicinity of the start codon [4]. The 5’ coding region of M9dH11 showed lower (more stable) folding energy that that of M5bG7 and EH-Ar and consequently higher propensity to partition to secondary structures (Fig 3B). To increase the folding energy in this initial region and disrupt secondary structures, the third base of five codons downstream of the start codon was exchanged to A which resulted in M9dH11_opt construct rendering the secondary structure propensity of this region (3^rd^ to 7^th^ codon, Fig 3B, shadowed area) in a range similar as for M5bG7 and EH-Ar; further downstream in the sequence the folding energies of the mRNA sequences of the EH variants differed but those are irrelevant for translation initiation [4]. All exchanges were synonymous and did not alter the amino acid sequence, but notably, they boosted the expression yield of the M9dH11_opt, and like wild-type M5bG7, a large fraction of it partitioned to the insoluble fraction (Fig 3C and 3D). However, at one hour induction the expression of M9H11_opt remained lower than those of M5bG7; comparable expression levels with M5bG7 were achieved when expressing M9dH11_opt for 2 h. Introduction of slow-translating regions at the domain boundaries (M9-LL_opt) increased the solubility to nearly 90% (Fig 3C and 3D). Notably, each attenuation sites alone (M9-L1_opt and M9-L2_opt) resulted in similar enhancement of the solubility (Fig 3C and 3D). The effect was not due to any increase in the mRNA expression level; the amount of mRNA for all M9dH11_opt variants remained the same (Fig 3E).

Taken together, these results clearly evidence that the solubility of heterologously expressed protein can be enhanced by adapting its translation profile to the tRNA pool of the expression host. Introducing slow-translating regions downstream of the domain boundaries greatly increased the solubility of a multi-domain protein.

## Discussion

**Fig 4:**
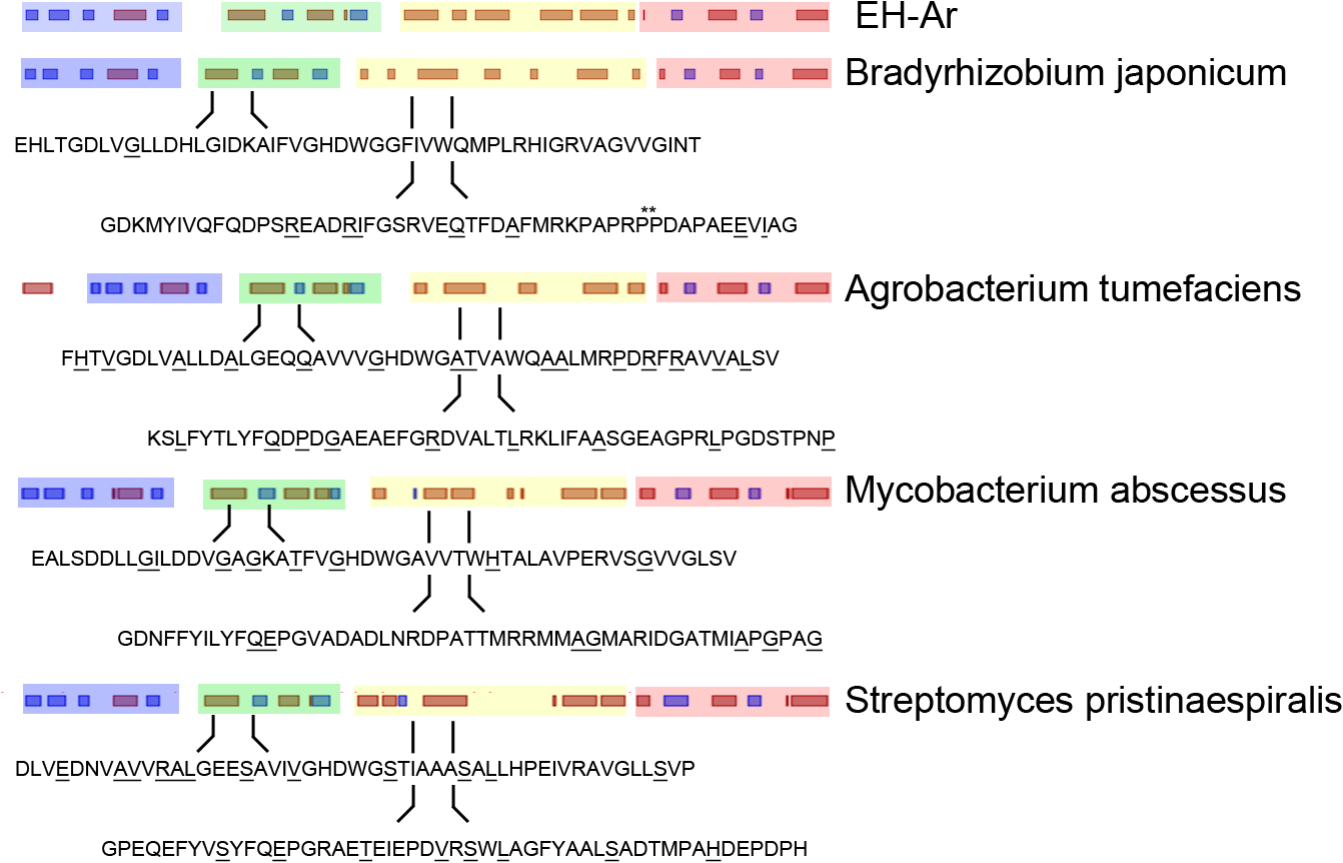
Overview of EHs from different species. Secondary structure alignment (β-sheets—blue bars, α-helices—dark red bars, uncolored empty space—linking structural elements) of EHs from different organisms [54]. The domains are color coded according to EH-Ar crystal structure (PDB 1EHY, Fig 1A). Downstream of the domain boundaries (20–70 aa), according to the identified slow-translating regions of EH-Ar (translation profile, Fig 1A), the amino acid sequences of each EH is shown and the amino acids encoded by rare codons are underlined. Rare codons are defined as the ten least used codons for each species.

Despite its importance in research (e.g. in structural analysis) and industrial applications, producing a protein in microbial hosts can be a challenging task and often needs individual optimization for each gene. Here, we suggest a rational, structure-guided design to modulate translation profiles that enhance solubility of a target protein in the bacterial host. By considering the co-translational hierarchical folding of the single domains in a heterologous expression system, we introduce slow-translating regions downstream of the putative domain boundaries to enhance the solubility of two EHs with metagenomc origin in E. *coli*. We used a protein family member (here EH-Ar) with solved crystal structure to delineate domain boundaries in EH homologs of unknown structure based on conserved secondary structure pattern. In the case of multidomain EH enzymes, our results clearly show that this strategy enhances the solubility of EHs of metagenomic origin up to 90%. The positions of slow-translating patches are chosen to allow a whole domain to be completely outside of the ribosome following a stretch that will be covered in the ribosomal tunnel. For the EHs this stretch is approximately 30 aa, but the length of the stretch protected in the ribosomal tunnel may vary (20–70 aa), and depends on the propensity of the nascent chain in this part to form extended or compact, α-helical, conformation [51]. Comparison of EHs from different organisms, expressed in a soluble form in their hosts [54], revealed that based on the corresponding codon usage rare codons are present in the regions down-stream (20–70 aa) of the domain boundaries (Fig 4). Even though the tRNA concentration of those organisms is unknown, for the majority of these codons the genomic tRNA copy number is low suggesting that those regions are most likely translated slower than the neighboring sequences within the domains. The common shape of the attenuation signature for many EHs in their natural hosts might be an additional selective force to preserve high fidelity of co-translational folding of conserved domains across species [42]. Thus, our approach of synchronizing the conserved natural translation profiles to the tRNAome household, or at least to the codon usage, of the expression host proves to be a successful strategy to enhance the yields of soluble protein.

Strikingly, the mere exchange of a single Leu to the synonymous CUA codon is sufficient to slow down nascent chain elongation in this region and enhance solubility of the protein product. The CUA codon is read by the rarest tRNA in *E*. *coli* [33] and is particularly useful due to this unique feature; even a single Leu codon might be sufficient to transiently attenuate the ribosomes [27]. If there is no Leu for synonymous variation in the region to be rendered slow-translating, substitutions of a few codons with their slow-translating counterparts should be considered. Usually groups of slow-translating codons, rarely consecutive but rather within a short sequence window of the mRNA elicit transient attenuation of the ribosome traffic [42]. However, changes in the coding sequence, even also of a single codon, may alter mRNA stability [55, 56]. In our case, the synonymous exchanges to convert Leu codons to slow-translating ones in both M5bG7 and M9dH11 caused none or subtle changes in the local mRNA folding energy which is mirrored by the equal mRNA levels between the M5bG7 and M9dH11 and their variants with synonymous exchanges. The efficiency of a nucleotide to modulate the local secondary structure propensity depends on the local sequence. Thus, while some single nucleotide substitutions may alter the local secondary structure [55, 56], others may remain indifferent for the secondary structure. In turn, to significantly influence the local folding energy, if this is desired, more than one nucleotide substitutions might be needed; to lower the folding energy in the vicinity of the start codon of M9dH11 we have undertaken five substitutions.

Importantly, for proteins expressed at a very low level, as in the case of M9dH11, it might be necessary to first optimize the efficiency of translation initiation before modulating solubility. Very low expression yields even with inefficient translation pattern may still result in soluble proteins as the concentration of a misfolded aggregation-prone species may remain under the critical concentration for initiating aggregation [57]. In line with this is the observation that expression at low induction regimes can result in functional folded proteins [31]. The structure of the initial 5’-coding sequence heavily influences initiation, i.e. ribosome loading, and consequently gene expression [4, 5]. Decreasing the propensity of M9dH11 to form secondary structure in the initial region by synonymous substitution of some codons in the 5’-coding region with their A-rich counterparts increases its total expression which in combination with translational pauses along the mRNA dramatically enhances soluble yields of the protein. Thus, various parameters in mRNA structure and sequence should be simultaneously optimized to enhance the production of active folded protein.

Variations in tRNA abundances among different organisms affect the translation pattern which consequently alters the expression of heterologous proteins. A rational design of synonymous substitutions to harmonize translation profiles is possible for only a handful of organisms; so far concentration of the full tRNA set has been determined for very few organisms, including *E*. *coli*, *B*. *subtilis* and *L*. *lactis* [33–35]. As a close proxy to harmonize translation profiles in the expression host, the codon usage [32, 58] or tRNA gene copies [59] can be used, although they do not precisely mirror the tRNA concentration [42]. Our structure-based approach is independent of the information on the tRNAome of the parental strain, but it requires at least one 3D structure of a homolog to identify the structural domains. Introduction of translational attenuation sites that delineate structural domains in otherwise smooth translation profiles remarkably increases the soluble expression. This adaptation of the translation profile to the tRNA abundances of the new host proves to be a successful strategy to enhance the yields of soluble, folded and active protein.
